# Diverse diets and low‐fiber, low‐tannin foraging preferences: Foraging criteria of Tibetan macaques (*Macaca thibetana*) at low altitude in Huangshan

**DOI:** 10.1002/ece3.9338

**Published:** 2022-10-04

**Authors:** Bowen Li, Wenbo Li, Chao Liu, Peipei Yang, Jinhua Li

**Affiliations:** ^1^ School of Resources and Environmental Engineering, Anhui University Hefei China; ^2^ International Collaborative Research Center for Huangshan Biodiversity and Tibetan Macaque Behavioral Ecology Anhui University Hefei China; ^3^ Key Laboratory of Animal Ecology and Conservation Biology Institute of Zoology, Chinese Academy of Sciences Beijing China; ^4^ School of Life Sciences, Hefei Normal University Hefei China

**Keywords:** fiber, food choices, low altitude, nutrient contents, tannin, Tibetan macaques

## Abstract

Nutrient composition and food availability determine food choices and foraging strategies of animals, while altitude and geographical location affect species distribution and food availability. Tibetan macaques (*Macaca thibetana*) have sophisticated foraging strategies as the largest species in *Macaca*. They are important in understanding the ecological evolution of the entire genus. However, the mechanism of food selection in Tibetan macaques at low altitudes remains unclear. In this study, we researched a wild Tibetan macaques group (Tianhu Mountain Group, 29 individuals) living in a low‐altitude area around Mt. Huangshan, Anhui Province, China. We used instantaneous scan sampling to observe these macaques' foraging behavior from September 2020 to August 2021. We recorded the dietary composition and food availability, compared the nutrient content of staple food and non‐food items, and analyzed the role of key nutrients in food selection. We found that Tibetan macaques forage on 111 plants belonging to 93 genera and 55 families. The food types included fruits (52.5%), mature leaves (17.0%), bamboo shoots (14.4%), young leaves (6.3%), flowers (4.5%), others (2.1%), stems (1.9%), and tender shoots (1.3%). Tibetan macaques forage for a maximum of 76 plant species during spring. However, dietary diversity was highest during summer (*H′* = 3.052). Monthly fruit consumption was positively correlated with food availability. Staple foods are lower in fiber, tannin, and water than non‐foods. In addition, the time spent foraging for specific foods was negatively correlated with the fiber and tannin content of the food. The results showed that Tibetan macaques' foraging plant species and food types were diverse, and their foraging strategies varied seasonally. Our findings confirmed the effect of nutrients on food choice in Tibetan macaques. We highlighted the important role of fiber and tannin in their food choices and suggested that the foraging behavior of Tibetan macaques is highly flexible and adaptive.

## Graphical abstract

Our study found that the foraging behavior of Tibetan macaques is highly flexible and adaptable. They prefer low‐fiber and low‐tannin foods when foraging for food. This dietary preference adapts to higher species richness in low‐altitude habitats, and may allow Tibetan macaques to maintain their large body size. We demonstrated the complexity of primate foraging behavior in terms of nutrition, food availability, and geographical distribution. The diet of primates should be defined comprehensively rather than simply as folivore or frugivore.

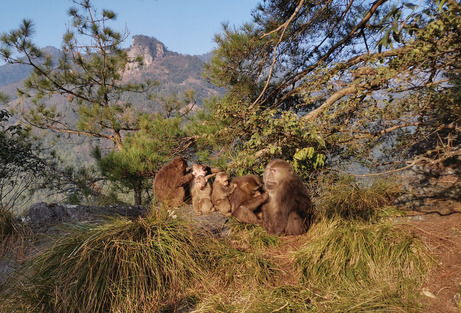

## 1 INTRODUCTION

Nutrient composition and food availability determine food choices and foraging strategies (Lambert & Rothman, 2015). Therefore, an appropriate diet is essential for animals. The dietary structure is a complex and variable combination of nutrients that has diverse functional implications for the consumer (DeCasien et al., 2017; Simpson & Raubenheimer, 2012; Vincze et al., 2022). Most natural foods do not provide a balanced diet; some have a surfeit of nutrients and a dearth of others (Raubenheimer et al., 2012). Therefore, animals must optimize their foraging strategies to adapt to temporal and spatial changes in the environment to improve their ecological adaptability.

Animal foraging strategies are based on finding and consuming essential nutrients while avoiding the adverse effects of plant secondary metabolites (Lambert, 2007; Simpson et al., 2004). Nutrition plays a critical regulatory role in species‐environment interactions (Grainger et al., 2020). Currently, the following five theoretical hypotheses in nutritional ecology exist for explaining nutrient foraging strategies. (1) The energy maximization hypothesis holds that animals seek to maximize their energy intake. In other words, total energy is the most important nutritional variable in animal foraging (Schoener, 1971); (2) The nitrogen (protein) maximization hypothesis states that animals choose food in the mode of nitrogen (protein) maximization (Mattson, 1980); (3) According to the regulation and avoidance hypothesis of plant secondary metabolites, animals generally avoid the intake of toxic secondary metabolites (Freeland & Janzen, 1974); while (4) according to the limitations on the intake of dietary fiber hypothesis, animal feeding is affected by the fiber content of plants (Milton, 1979); (5) Finally, the nutrient balance hypothesis holds that animals balance their nutrition by mixing different foods with different nutrients, rather than maximizing the availability of one nutrient (Raubenheimer & Simpson, 2004). However, these hypotheses do not apply to all primates. Foraging behavior in different primate species is specific and complex, and requires detailed analysis for different situations.

Several studies have shown that tropical primates have a complex diet. Some primates are frugivorous species, like Cao Vit gibbon (*Nomascus nasutus*) (Ma et al., 2017), Lar gibbon (*Hylobates lar*) (Subramaniam, 1981), Crab‐eating macaque (*M. fascicularis*) (Ménard, 2004), and Pig‐tailed macaque (*M. nemestrina*) (Ménard, 2004), while others are folivorous or omnivorous species, such as Indri (*Indri indri*) (Britt et al., 2002), eastern black and white colobus (*Colobus guereza*) (Fashing et al., 2007), and black howler monkey (*Alouatta pigra*) (Amato & Garber, 2014). Tropical areas are rich in plant species and have high plant productivity. Therefore, primates in tropical regions have more food choices to meet their needs. Primates observed in subtropical and temperate latitudes have complex diets. They more often belong to folivorous or omnivorous, such as Taihangshan macaque (*M. mulatta tcheliensis*) and Barbary macaque (*M. sylvanus*) (Cui et al., 2019; Hanya et al., 2011). Plant productivity is lower at temperate latitudes than at tropical latitudes (Cramer et al., 1999). The temperate areas are dominated by evergreen forests and fruit availability is relatively low (Hanya & Chapman, 2013). Therefore, the same species may show different foraging preferences in different climate zones. For example, the Assamese macaque (*M. assamensis*) in tropical forests forages more fruits, whereas those in temperate forests forage more leaves (Ahsan, 1994; Heesen et al., 2013).

Altitude also affects food resource distribution. Previous studies have shown that temperature and biodiversity change with altitude. Higher altitudes are characterized by lower temperatures, lower plant diversity, and fewer fruits (Glowacka et al., 2017; Kӧrner, 2003). Primates adapt to these changes by altering their foraging strategies. For example, in Rhesus macaque (*M. mulatta*), the number of plant species consumed ranges from 102 species at 225 m, to 35 species at 2360 m (Feeroz, 2012; Goldstein & Richard, 1989; Peng et al., 2008; Tang et al., 2016; Tomar & Sikarwar, 2014). Macaques in lowland forests feed mainly on fruit and animal matter, whereas populations in alpine forests feed more on foliage and items such as bark and fungi (Tsuji et al., 2013).

Proteins and carbohydrates play decisive roles in primate food choices (Ganzhorn et al., 2017; Li et al., 2020; Ma et al., 2017). Proteins are crucial functional substances in living organisms and are the only source of dietary nitrogen. Many primates tend to forage for foods high in protein and low in fiber (Ganzhorn et al., 2017), as evidenced by *M. assamensis* (Li et al., 2020), White‐footed sportive lemur (*Lepilemur leucopus*) (Dröscher et al., 2016), *C. guereza* (Fashing et al., 2007) and *N. nasutus* (Ma et al., 2017). This is because the fiber content can affect food palatability and reduce digestion efficiency (Barboza et al., 2010). Carbohydrates are a primary source of energy in animals. Many primates enjoy fruit because of their high sugar content. Studies of captive Western lowland gorillas (*Gorilla gorilla gorilla*) and Chimpanzees (*Pan troglodytes*) show a preference for foods with high sugar content, despite their different body sizes (Remis, 2002). Mountain gorillas (*G. beringei*) in Uganda also show a foraging preference for high‐carbohydrate fruits (Rothman et al., 2006). Lipids are also an important source of energy for the organism, which can provide twice the energy of sugar and protein per gram (Righini et al., 2015). Lipids also provide essential fatty acids and are involved in the synthesis of other substances (Raubenheimer et al., 2012). Therefore, fat has an important effect on the food choices of animals. For example, in the study of black howler monkeys, there was a significant positive correlation between food choices and lipids during cold and dry seasons (Righini et al., 2015).

The Tibetan macaque is endemic to China and is classified as near‐threatened by the International Union for Conservation of Nature's (IUCN) Red List in 2021. They have the largest body size in the *Macaca* genus. They live in temperate and subtropical mountainous forest environments and have adapted to cold climates. They are mostly found at altitudes above 600 m and up to 2400 m and exhibit rich and varied social behavior (Li & Kappeler, 2020; Li et al., 2020; Wada et al., 1987). Body size constrains primate food selection (Ford & Davis, 1992). Tibetan macaques must have sophisticated foraging strategies to ensure sufficient energy for maintaining their large body size and adapting to cold environments. In previous studies, the Tibetan macaque was found to be a folivore primate (Thierry, 2007; Zhao, 1996). Studies have found that Tibetan macaques feed on 50 plant species at Mt. Huangshan and 196 at Mt. Emei, with significant seasonal differences (You et al., 2013; Zhao et al., 1991). However, these studies did not delve into the role of nutrient composition in foraging selection. They did not analyze the foraging and nutritional strategy of macaques. Detailed data on the nutritional strategy of Tibetan macaques are not yet available. Further study of the foraging choice of macaques under natural conditions is necessary to reflect the foraging strategy more truly and reveal their adaptability to different habitats. This study will fill the gaps in foraging nutrition strategies in Tibetan macaques. It is beneficial for proposing more effective measures for protecting Tibetan macaques and has some significance for revealing the foraging strategy of the whole *Macaca* genus.

In 2018, we discovered a group of wild Tibetan macaques in a low‐altitude area (200–600 m) near the east gate of Mt. Huangshan and started tracking them in 2019. As a typically high‐altitude dwelling species, we expect low‐altitude Tibetan macaques to forage differently than those at higher altitudes, yet we lack data for low‐altitude Tibetan macaques. In this study, we aim to (1) identify the dietary composition and seasonal changes in Tibetan macaques and analyze the relationship between foraging choice and food availability, (2) measure and compare the nutrient content of food and non‐food, and (3) analyze the effects of different nutrients on the feeding time of Tibetan macaques. We expected to determine the Tibetan macaques' diet and the nutritional factors that influence their foraging choices by testing the following predictions:

- Significant differences exist in the number of plant species foraged by the same primate species living in different spatial and temporal environments. Areas at lower elevations have higher biodiversity and resource availability. Therefore, we predict that Tibetan macaques at lower altitudes will forage for as many different plant species as possible and that there will be seasonal differences in foraging plants.
- Due to the higher productivity of ecosystems at lower elevations, we predict that Tibetan macaques tend to prefer high‐quality foods and will forage more for fruit when fruit abundance is higher.
- Based on the nitrogen (protein) maximization model, plant secondary metabolites avoidance model, and the limitation of the dietary fiber model, we predicted that Tibetan macaques in low‐altitude habitats would forage for more high‐protein, low‐fiber, and low‐tannin foods when faced with relatively abundant food resources.
- Tibetan macaques require more energy to meet their physical needs as the largest primate of the genus *Macaca*. We predict that Tibetan macaques will select foods high in sugar and fat.

## 2 METHODS

### 2.1 Study site and animals

This study was conducted in the Niejiashan area (30°12′N, 118°27′ E, 200–600 m above sea level) at the junction of Mt. Huangshan and the Tianhu Nature Reserve, in Anhui Province, China (Figure 2). The research base was established in 2018 by Huangshan Biodiversity and the Tibetan Macaque Behavioral Ecology International Research Center. This area has a subtropical monsoon climate zone and four distinct seasons. The total annual rainfall is 2639.4 mm, and the average annual temperature is 15.5°C, with a maximum of 38.1°C and a minimum of −13.1°C. Following Li et al. (2021), the four seasons were defined according to the monthly average temperature. An average temperature greater than 22°C is classified as summer (June–September), <10°C as winter (December–February), and 10–22°C as spring (March–May) and autumn (October–November) (Li et al., 2021).

**FIGURE 1:**
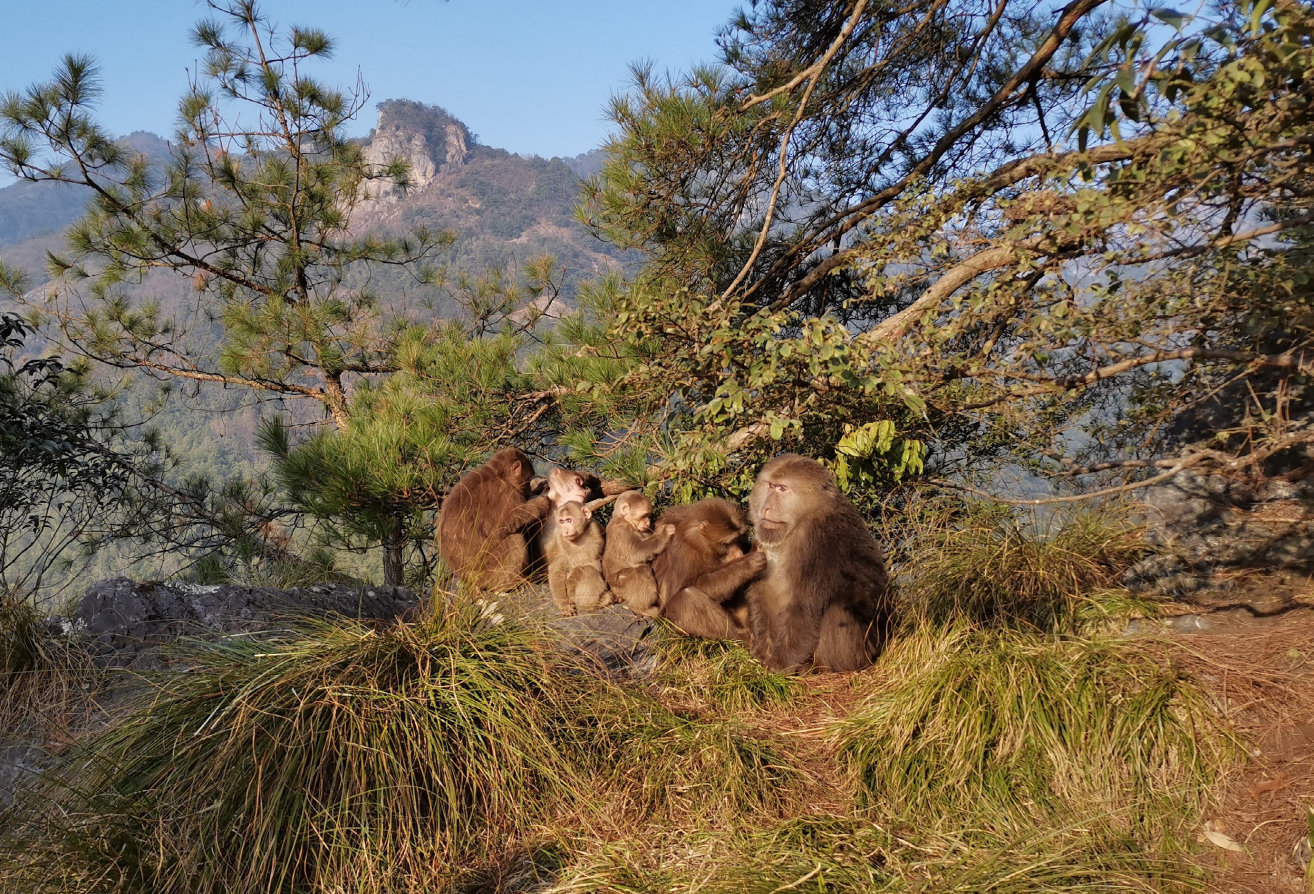
Tibetan macaques (*Macaca thibetana*) (from left to right are four juveniles and two adult males)

We studied a group of 29 wild Tibetan macaques (Figure 1), including 5 adult males, 8 adult females, 10 juveniles, and 6 infants. We can distinguish age according to body size and the shade of hair color. These macaques spent extensive time at altitudes below 600 m.

**FIGURE 2:**
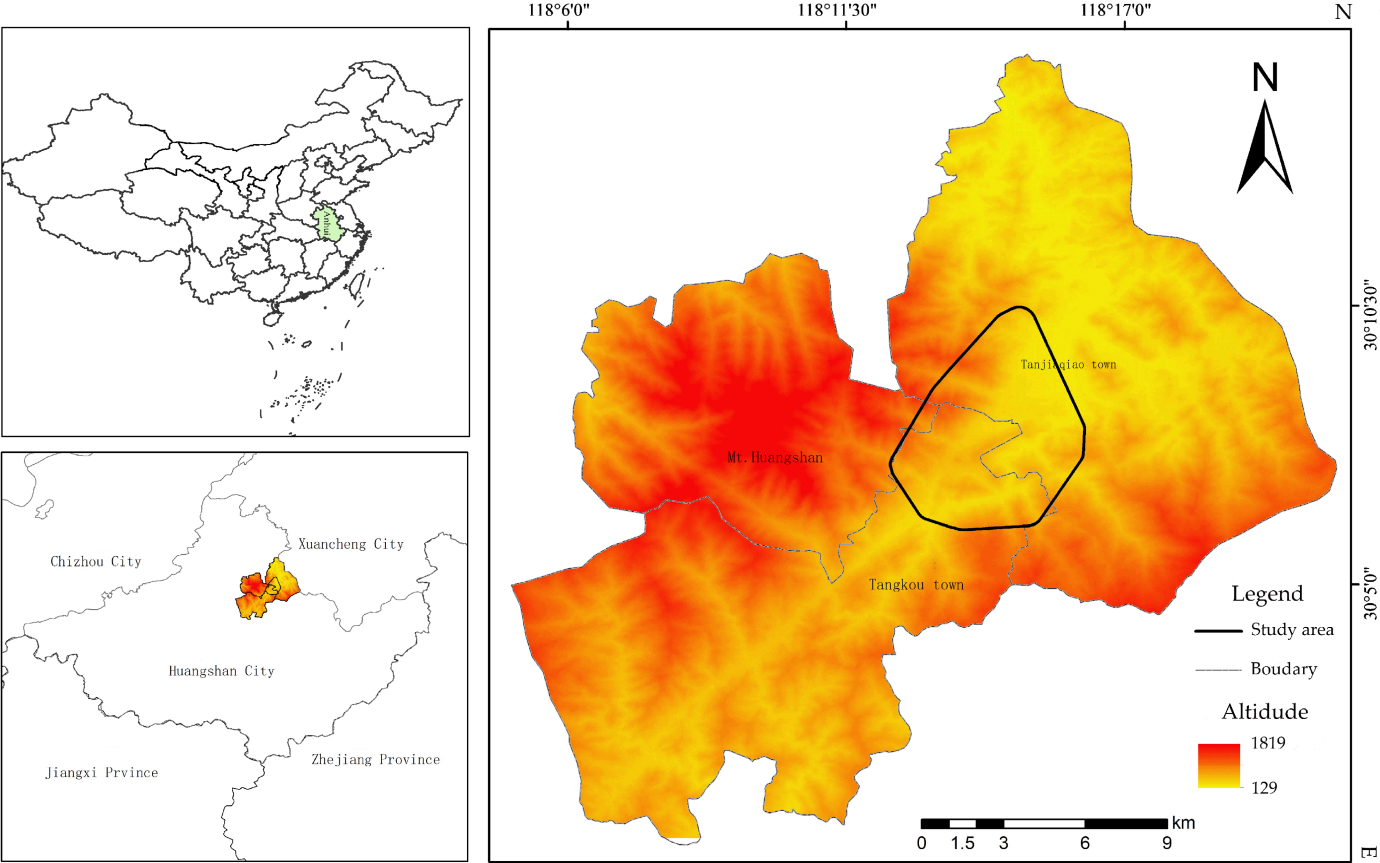
Study site (the study site was situated at the boundaries of the Huangshan Mountains and Tianhu nature reserve. The range of the black coil shown here is the home range of the Tibetan macaque).

**FIGURE 3:**
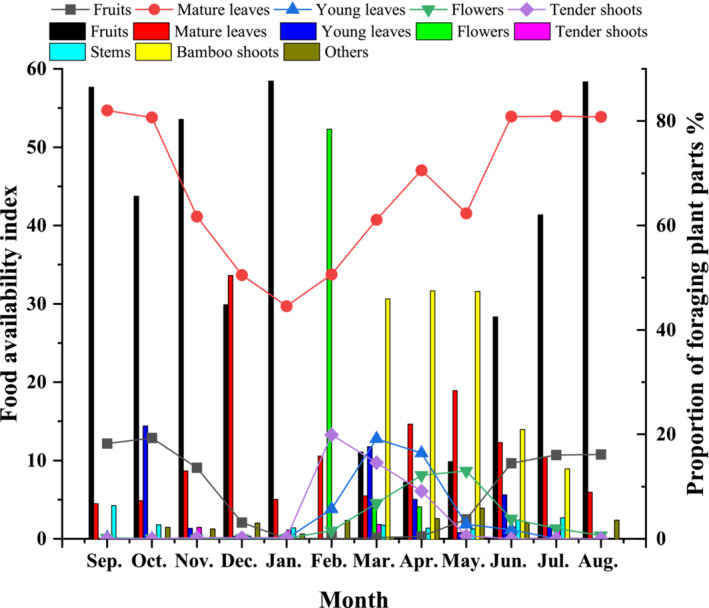
Food Availability Index (FAI) for fruits, mature leaves, young leaves, flowers, and tender shoots in the study site (line graph) and changes in food types of Tibetan macaques foraging in different months (Bar graph) (September 2020 to august 2021).

**FIGURE 4:**
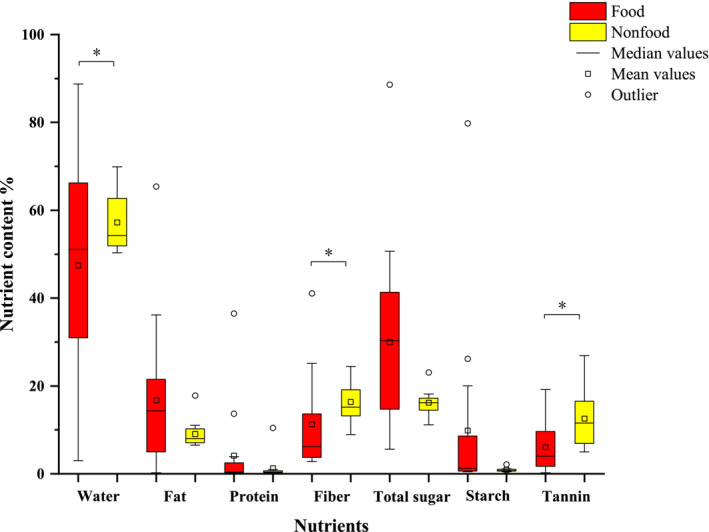
Comparison of nutritional components among food and non‐food (food: *N* = 15; non‐food: *N* = 12. χ^2^ test: *p < .05).

### 2.2 Data collection

#### 2.2.1 Behavioral data collection

The fieldwork began in August 2019 and ended in August 2021. We set up 30 automatic return infrared cameras (UML4, UOVISION) in the Tibetan macaques' habitat to identify the movement of the monkeys and to search for and track the monkeys based on those positions. Owing to the impact of COVID‐19, the data we collected were discontinuous during the first year. After a year of tracking and habituation, we had a home range for the group and were able to keep the data continuous.

We arrived at the Tibetan macaque's sleep site every morning before 6 o'clock. After locating the macaques, we used our phones' Liangbulu (Shenzhen Liangbulu Information Technology Co., Ltd.) GPS software to collect the group's geographical location. We then observed their behavior until they disappeared for 30 min or entered the sleeping place in the evening. We obtained continuous data from September 2020 to August 2021 and analyzed these data.

We used the instantaneous scanning sampling method to collect data (Altmann, 1974), with each scan lasting 5 min and a 5‐min interval between scams. We observed Tibetan macaques using binoculars (PROSTAFF7, Nikon) at distances of 20–100 m and used a voice recorder (ICD–PX470, SONY China) to record the data. To avoid sampling bias for specific individuals, as many individuals as possible were scanned from left to right at each sampling time. We observed each individual for 5 s during each scan and recorded their primary behaviors (classified as resting, moving, feeding, socializing, etc.). When individuals ate, we recorded the species and parts of the plants they ate, including mature leaves, young leaves, flowers, fruits, stems, tender shoots, and others. Because the foraging frequency of bamboo shoots was relatively high, and classification is complicated, bamboo shoots were listed separately as a food type. We also used ad libitum sampling to record the species of food plants between scans to obtain the diet composition (Altmann, 1974) but these data were not used for analysis. During the study period (September 2020 to August 2021), we obtained 32,914 individual records from 3034 scans, with an average of 10.85 individuals per scan (Table 1).

**TABLE 1:** Data acquisition during the study period

Month	Numbers of scan	Observation days per month
September	72	5
October	296	13
November	261	9
December	399	8
January	456	7
February	90	5
March	372	7
April	332	9
May	359	9
June	184	6
July	134	6
August	79	4
Total	3034	88

#### 2.2.2 Samples collection

We collected 81 samples (three samples per item) from 27 items of 23 plants, including 15 main food types and 12 dominant non‐food species in the environment (Table 2). To obtain the data on dominant vegetation species in the Tibetan macaques' home range, we set 33 20 × 20 m quadrats and 64 × 100 m transect zones according to the elevation gradient in the macaque home range from September 2019 to November 2019. The trees, shrubs, herbs, and vines were counted and identified (Li et al., 2021). We calculated the proportion of each food item in all feeding records, defining foods that accounted for 1% or more as important. When we observed macaques foraging for a certain plant, we collected 500 g from the same plant after the individuals left. Due to the complexity of field sampling, we were unable to collect important food samples from dangerous terrains. Samples of dominant species were collected in the same manner. Samples were then prepared for nutritional analysis, described below.

**TABLE 2:** Food and non‐food samples used for nutritional analysis

Species	Plant parts	Food/non‐food
*Phyllostachys edulis*	B	Y
*Castanopsis eyrei*	F	Y
	L	N
*Pinus massoniana*	F	Y
*Stauntonia brachyanthera*	F	Y
*Celtis sinensis*	F	Y
*Eurya japonica*	F	Y
	L	Y
*Millettia dielsiana*	L	Y
*Cyclobalanopsis glauca*	F	Y
	L	N
*Lindera glauca*	F	Y
*Rosa laevigata*	F	Y
*Neyraudia montana*	S	Y
*Diospyros kaki var. silvestris*	F	Y
*Lithocarpus brevicaudatus*	F	Y
	L	N
*Symplocos stellaris*	L	Y
*Castanopsis sclerophylla*	L	N
*Cunninghamia lanceolata*	L	N
*Litsea coreana*	L	N
*Quercus serrata*	L	N
*Phoebe sheareri*	L	N
*Alniphyllum fortunei*	F	N
*Liquidambar formosana*	F	N
*Lindera aggregata*	L	N
*Camellia fraterna*	L	N

Abbreviations: B, bamboo shoots; F, fruit; L, leaf; S, stem; Y, species/items used for food by Tibetan macaques; N, species/items not used for food by Tibetan macaques.

#### 2.2.3 Food availability assessment

Based on the results of the first year (August 2019 to August 2020), 35 plants were selected for phenological monitoring. We randomly selected 10 plants of each species from the main range of Tibetan macaques and monitored them in the middle of each month. We recorded the growth of the mature leaves, young leaves, flowers, fruits, and tender shoots. Quantitative statistics were performed by estimating the proportion of individual food types to the canopy volume of the plants. To facilitate the estimation and ensure the relative accuracy of the value, we divided the sub‐ratio into five levels: 0 < 1%, 1 = 1%–25%, 2 = 26%–50%, 3 = 51%–75%, and 4 > 75%. The following formula was used to calculate the availability of individual foods per month (Silver et al., 1998).

$$A_{i}=C_{i}\times \bar{V}$$

where $C_{i}$ is the sum of the thorax height area of the instant feeding species *i* and $\bar{V}$ is the mean ratio of *i* to the crown volume per tree in a certain month. The sum of the same food types is the availability of this food type.

### 2.3 Nutritional analysis

All samples were weighed to determine the fresh weight (*W*_*t*_). The samples were then baked in a 105°C thermostatic drier box (101–3BS, LICHEN) for 15 min and dried at 80°C until a constant weight (*W*_*c*_). The gravimetric method was used to determine the moisture content of the samples (Standardization Administration of the People's Republic of China, 2011a):

$$\text{Water}\;\left(\%\right)=\frac{W_{t}-W_{c}}{W_{t}}\times 100\%$$

After calculating the moisture content, we ground the sample and passed it through a 40 mesh sieve. The samples were sealed and brought back to the biochemical laboratory of Anhui University for determining the content of other nutrients. Protein content was determined using the Coomassie Brilliant Blue G‐250 (Bradford, 1976). Total sugar was measured using the 3,5‐dinitrosalicylic acid assay (Dubois et al., 1956). Starch was measured using the enzymatic hydrolysis method (Standardization Administration of the People's Republic of China, 2016). Fiber content was measured using anthrone colorimetry (Li et al., 2018). Crude fat content was determined using the Soxhlet extraction method (Standardization Administration of the People's Republic of China, 2003). Tannin content was determined using the colorimetric method (Standardization Administration of the People's Republic of China, 2011b).

### 2.4 Data analysis

Following Shaffer (2013) and Huang et al. (2015), we first divided the number of individuals foraging for a particular food in each scan by the number of individuals recorded in that scan and then divided this value by the proportion of individuals engaged in feeding activity in the scan to indicate the percentage of foraging time for a particular food. The average of all scans over an hour represents the time spent foraging for a specific food per hour. To correct deviations that may result from uneven scanning records throughout the day, we averaged the hourly data to represent the daily feeding time of certain foods. The average daily data were used to indicate the monthly feeding time on specific food items. The average monthly data were then used to determine the time of the year when certain foods were consumed.

We used the Shannon–Wiener diversity index to express the dietary breadth of Tibetan macaques, $H^{\prime }=-\sum_{i=1}^{n}\mathrm{Pi}\ln\mathrm{Pi}$ (*Pi* is the percentage of feeding records of plant species *i*) (Huang et al., 2015). We tested all variables using a one‐sample Kolmogorov–Smirnov test to examine normality. We used Pearson's and Spearman's correlations to examine the relationship between consumption of different food types and the relationship between food type consumption and food availability. All tests were two‐tailed, with a significance of .05.

To improve linearity, logit (X) transformation was applied to variables expressed as percentages, such as water, protein, fiber, total sugar, starch, crude fat, and tannin. Logit (X + 0.00001) transformation was applied to feeding time because the original data of feeding time for non‐food species were 0 (Warton & Hui, 2011). We constructed a generalized linear mixed model (GLMM) to examine the differences in nutrient composition between food and non‐food. In the model, the contents of water, fat, protein, fiber, sugar, starch, and tannin were set as the response variable, respectively, plant type (food or non‐food) was set as a fixed factor, plant parts and plant species were set as random factors. We then compared the models with and without fixed factors using anova (analysis of variance) for determining the influence of fixed factors on the dependent variables. When *p* < .05, the fixed factor significantly affected the goodness of fit of the model, indicating that there were significant differences in response variables between food and no‐food.

We constructed GLMMs to study the effects of different nutrients on feeding time. We set the annual feeding time of plants as the response variable, the nutrient composition as fixed factors, and the plant species and parts as random factors in the model. We obtained 2^7^–1 = 127 models from all possible combinations of the 7 fixed factors, and multi‐model reasoning and model averaging were conducted based on information theory (Akaike's information criterion corrected for small sample sizes, AICc). In the model construction process, 7 nutrients (water, fiber, fat, protein, total sugar, starch, tannin) were screened into the model and the “glmulti” function was used to screen all possible models and select the optimal model. The models were sorted according to their AICc values, and the model with the lowest AICc value was the model with the highest ranking. We selected the model with the highest support, and the AICc value difference between each model and the model with the highest ranking was ΔAICc ≤ 2. We calculated the Akaike weight (*W*_*i*_) of each model with the highest support. In model averaging, the relative importance (*W*_*ip*_) of each predictive variable is assessed by summing the W_i_ values of all models including specific variable (Burnham & Anderson, 2002). The GLMMs were constructed using the *lmer* function of the *lme4* package (Bates et al., 2015); the model averaging was performed using *dredge* and *model.avg* function of the *MuMIn* package (Bartoń, 2019). All analyses were conducted using R v4.1.2. All tests were two‐tailed, with a significance level of .05.

## 3 RESULTS

### 3.1 Dietary composition of Tibetan macaque

A total of 111 forage plant species belonging to 55 families and 93 genera were recorded during field observations. The food types included fruit (52.5%), mature leaves (17.0%), bamboo shoots (14.4%), young leaves (6.3%), flowers (4.5%), others (2.1%), stems (1.9%), and tender shoots (1.3%) (Table 3). According to the feeding records from September 2020 to August 2021, 16 plants were defined as important food sources, accounting for 80.58% of the total consumption of Tibetan macaques (Table 3).

**TABLE 3:** Percentage of foraging frequency for different food types in Tibetan macaques

Food types	Species numbers	Species	Ratio of foraging frequency %	Total %
Fruits	1	*Castanopsis eyrei*	15.38	52.50
	2	*Pinus massoniana*	14.38	
	3	*Stauntonia brachyanthera*	6.44	
	4	*Cyclobalanopsis glauca*	2.07	
	5	*Rosa laevigata*	1.94	
	6	*Celtis sinensis*	1.80	
	7	*Cerasus serrulata*	1.73	
	8	*Ulmus pumila*	1.43	
	9	*Eurya japonica*	1.33	
	10	*Actinidia rubricaulis var. coriacea*	1.02	
	11	*Litsea pungens*	0.99	
		Others	3.99	
Mature leaves	12	*Phyllostachys edulis*	5.43	17.00
	13	*Millettia dielsiana*	2.58	
	9	*Eurya japonica*	2.15	
	2	*Pinus massoniana*	0.26	
	7	*Cerasus serrulata*	0.15	
		Others	6.43	
Bamboo shoots	12	*Phyllostachys edulis*	11.02	14.4
	14	*Phyllostachys sulphurea var. viridis*	3.38	
Young leaves	15	*Salix chaenomeloides*	2.24	6.30
	6	*Celtis sinensis*	1.11	
		Others	2.95	
Flowers	6	*Celtis sinensis*	2.52	4.50
		Others	1.98	
Stem	16	*Neyraudia montana*	1.14	1.90
		Others	0.76	
Tender shoots	4	*Cyclobalanopsis glauca*	0.34	1.30
	6	*Celtis sinensis*	0.20	
		Others	0.76	
Others			2.10	2.10
Total (%)			100	100

*Note*: The numbers 1–16 represent the proportion of foraging frequency of important food for Tibetan macaques. Others indicate the foraging food except for important foods.

### 3.2 Seasonal variations in diet

The number of food species consumed and dietary diversity (Shannon–Wiener index, *H*′) differed seasonally. Tibetan macaques forage for the greatest number of plant species in spring (*n* = 76) and the fewest in autumn (*n* = 46). However, dietary diversity was highest in summer (*H*′ = 3.052) and lowest in autumn (*H*′ = 2.169) (Table 4). It is worth noting that there are one or two plants that Tibetan macaques fed more heavily on during each season. For example, bamboo shoots of *Phyllostachys edulis* were fed on in spring (37.2%), *Eurya japonica* in summer (16.9%), *Stauntonia brachyanthera* (33.1%), and *Castanopsis eyrei* (26.9%) in autumn, and the fruits of *Pinus massoniana* (31.6%) and *Castanopsis eyrei* (20.5%) in winter.

**TABLE 4:** Number of food species and dietary diversity of Tibetan macaques in different seasons

Season	Number of food species	Dietary diversity (*H*′)
Spring	76	2.626
Summer	60	3.052
Autumn	46	2.169
Winter	58	2.395

The food types consumed by Tibetan macaques also varied seasonally. Fruit foraging peaked from September 2020 to November 2020, flowers and young leaves mainly from February 2021 to March 2021, and bamboo shoots mainly from March 2021 to May 2021 (Figure 3). The consumption of fruits and mature leaves (*r*_s_ = −.650, *N* = 12, *p* < .05), fruits and flowers (*r*_s_ = −.819, *N* = 12, *p* = .001), and fruits and bamboo shoots (*r*_s_ = −.632, *N* = 12, *p* < .05) was negatively correlated. Phenology also changed significantly between months (Figure 3). The availability of fruit peaked from September 2020 to October 2020, flowers and young leaves from March 2021 to May 2021, shoots in February 2021, and mature leaves remained relatively stable throughout the year. When analyzing the food availability and foraging choice of Tibetan macaques, we found that monthly fruit consumption was positively correlated with availability (*r* = .651, *N* = 12, *p* < .05).

### 3.3 Comparison of nutrient content between food and non‐food items

Analysis of the nutritional content of both food and non‐food revealed that the foods were less rich in water (*χ* ^2^ = 8.6333, *df* = 1, *p* = .003), fiber (*χ* ^2^ = 5.5921, *df* = 1, *p* = .018), and tannin (*χ* ^2^ = 5.8553, *df* = 1, *p* = .0155). However, there was no difference in contents of fat (*χ* ^2^ = 0.2188, *df* = 1, *p* = .6399), protein (*χ* ^2^ = 0.1164, *df* = 1, *p* = .733), starch (*χ* ^2^ = 2.4459, *df* = 1, *p* = .1178), and total sugar (*χ* ^2^ = 2.9611, *df* = 1, *p* = .0853) (Table 5, Figure 4).

**TABLE 5:** GLMM model results for explaining the differences in nutrient contents of food and non‐food species

Response variable	Explanatory variable	Estimated values	Standard error	*t*
Water	Intercept	0.19155	0.10110	1.895
	Food species	−0.32186	0.06638	−4.848
Fat	Intercept	−0.89719	0.14850	−6.041
	Food species	−0.09671	0.17596	−0.550
Protein	Intercept	−2.226	0.3122	−7.132
	Food species	−0.00004	0.2717	0.000
Fiber	Intercept	−0.72482	0.08964	−8.085
	Food species	−0.30137	0.12027	−2.506
Total sugar	Intercept	−0.7201	0.1181	−6.098
	Food species	0.2725	0.1584	1.720
Starch	Intercept	−2.0408	0.2582	−7.903
	Food species	0.3735	0.2784	1.342
Tannin	Intercept	−0.9533	0.1274	−7.485
	Food species	−0.4525	0.1443	−3.135

### 3.4 Effects of nutrients on feeding time

The two most supported models (ΔAICc ≤ 2) included four variables: fiber, starch, total sugar, and tannin. The Akaike weight (*W*_*i*_) values of the two most supported models were 0.18 and 0.07, respectively (Table 6).

**TABLE 6:** The top two linear regressions models (lm) (ΔAICc ≤ 2) investigating the effects of nutrient content on the feeding effort of Tibetan macaques.

Model	*df*	Log‐likelihood	AIC_c_	ΔAIC_c_	*W*_*i*_
Fiber + total sugar + tannin	7	−39.50	98.90	0.00	0.18
Fiber + starch	6	−42.30	100.79	1.89	0.07

Abbreviations: AICc, Akaike's information criterion corrected for small sample sizes; ΔAICc, the difference between a specific model and the most high‐ranked one; *W*_*i*_ (Akaike weights), the probability that a model is optimal given the particular set of models considered.

Model averaging showed that fiber (*W*_*ip*_ = 0.74) and tannin (*W*_*ip*_ = 0.59) were significant factors affecting feeding time in Tibetan macaques (Table 7). The model‐averaged 95% confidence intervals of these two predictors never overlapped with zero. Feeding time decreased (*β* < 0) with increasing fiber and tannin. Although total sugar and starch were included in the two highly supported models (ΔAICc ≤ 2; Table 6), their model‐averaged 95% confidence interval of the regression coefficient (*β*) contained zero. Other factors including water, protein, and fat had comparatively minor effects (*W*_*ip*_ = 0.1–0.21), and the model‐averaged 95% confidence intervals of these predictors overlapped with zero.

**TABLE 7:** Summary of model averaging based on lm models using nutrient factors to explain the feeding time of Tibetan macaques on specific food.

Variable	*β*	SE	*z*	*p*	95% CI (lower to upper)	*W*_*ip*_
Fiber	**−2.0556**	**0.7776**	**2.506**	**.0122**	**−3.663 to −0.448**	**0.74**
Total sugar	1.3713	0.8544	1.542	.1232	−0.372 to 3.115	0.53
Tannin	**−1.4093**	**0.6142**	**20.187**	**.0288**	**−2.673** to **−0.146**	**0.59**
Starch	0.9430	0.6592	1.379	.1679	−0.397 to 2.283	0.4
Water	−0.6868	0.7084	0.923	.3561	−2.145 to 0.771	0.21
Fat	−0.2974	0.7517	0.381	.7034	−1.828 to 1.234	0.14
Protein	−0.3914	0.4769	0.778	.4363	−1.377 to 0.594	0.1

*Note*: Model‐averaged 95% confidence interval excluded zero value are shown in bold.

Abbreviations: 95% CI, the 95% confidence intervals for *β*; *W*_*ip*_, relative variable importance; *β*, model‐averaged regression coefficients.

## 4 DISCUSSION

Dietary composition and nutrition are essential factors for understanding foraging strategies in primates. We studied the foraging strategies of Tibetan macaques in low‐altitude habitats by analyzing their diets and the effects of nutrients on their foraging choices. The results presented 111 species of forage plants for Tibetan macaques living in the low‐altitude area of Mt. Huangshan. In addition, fruits account for 52.5% of the foraging food, indicating that Tibetan macaques in low‐altitude areas are highly frugivorous primates. The choice and types of food species gradually changed throughout the year, and monthly fruit consumption was positively correlated with food availability. Considering food choices, Tibetan macaques preferred plants that were low in fiber and tannin content. Our study sheds light on the nutrition‐based foraging strategies of Tibetan macaques and provides new insights into foraging strategies and ecological adaptations of other primates.

Our study found that Tibetan macaques at low altitudes forage for more unique plant species than Tibetan macaques at high altitudes in the same area (54 species, Wang, 2008; 50 species, You et al., 2013), and the number of food species is different in season. This is consistent with our prediction i. Altitude differences can lead to differences in species richness and food availability. Studies have shown that temperature and biodiversity change with altitude, with resources being more abundant at lower altitudes (Kӧrner, 2003). In this study, Tibetan macaques foraged heavily for foods not recorded in studies at high altitudes, such as *Stauntonia brachyanthera*. The number of species of food plants of macaques distributed on Mt. Emei (1260–2100 m above sea level) was higher than those in this study (196 species, Zhao et al., 1991), which may be due to the difference in geographical locations. Mt. Emei has a high species richness. The vegetation types include evergreen broad‐leaved forest, evergreen deciduous broad‐leaved mixed forest, and evergreen coniferous forest (Zhao et al., 1991). All this provides a rich food choice for the Tibetan macaques on Mt. Emei. Our results suggested that the foraging strategies of Tibetan macaques are highly flexible and adaptable and that both time and space affect macaques foraging choices. Their food intake changed with environmental resources, thereby achieving higher adaptability. Similar results have been found in *Indri indri*, *Macaca*, and *Colobinae*, wherein differences in geographical location and altitude led to differences in diet composition (Britt et al., 2002; Tsuji et al., 2013). Studies on Golden monkey (*Cercopithecus mitis kandti*) found that they also adjust their diet based on habitat and food availability (Tuyisingize et al., 2021). Previous studies have defined Tibetan macaques as folivore primates (Thierry, 2007; Wang, 2008; Zhao, 1996), but the findings of the current study provide a different perspective. We found that fruit feeding in Tibetan macaques is influenced by environmental food availability. When the fruit is plentiful, macaques reduce the consumption of other plant parts. This is consistent with our prediction ii. Owing to the effect of cold climates, fruit richness in high‐altitude areas is lower than that in low‐altitude areas (Glowacka et al., 2017; Kӧrner, 2003). Studies on *G. beringei beringei* established that elevation can lead to dramatic changes in diet, with more fruits consumed at lower altitudes than at higher altitudes (Ganas et al., 2004). The time spent foraging for fruits in baboons also decreases with elevation (Hill & Dunbar, 2002). This suggests that a broad dietary strategy cannot accurately classify primate feeding habits. Therefore, our results suggest that food availability is a crucial factor that affects the diet of Tibetan macaques. Some primates cannot be directly classified as folivores or frugivores.

Analysis of the nutritional content of food and non‐food items revealed that the contents of fiber, tannins, and water in foods were lower than those in non‐food items. The foraging choice of macaques was consistent with the dietary fiber restriction hypothesis and secondary metabolite avoidance hypothesis, in line with our prediction (iii). Fibers affect digestion efficiency and reduce food palatability (Barboza et al., 2010). Therefore, many primates avoid foods that are high in fiber. For example, red colobus monkeys (*Procolobus badius*), *C. guereza*, and *N. nasutus* show high selectivity for foods with low fiber content (Chapman & Chapman, 2002; Fashing et al., 2007; Ma et al., 2017). Tannins can bind to proteins, thereby affecting the digestibility of food and nutrient absorption (Wink, 2003). Studies on Bornean orangutans (*Pongo pygmaeus*), *C. guereza*, and *M. assamensis* have found that these primates avoid tannin‐rich foods (Fashing et al., 2007; Leighton, 1993; Li et al., 2020). Tibetan macaques chose plants with low fiber and tannin contents in this study. As mentioned above, Tibetan macaques foraged more fruits in this study. Fruit tends to have lower fiber and tannin content than mature leaves and other plant parts, which partly explains the low fiber and tannin content in their diet. Water is essential in the processes of thermoregulation and many primates choose juicy foods to meet their water requirement (Li et al., 2020). However, the water content in Tibetan macaques' food items was lower than that in non‐food items. There are abundant surface water resources in the habitat of Tibetan macaques, and small streams or reservoirs can be seen everywhere. Tibetan macaques have been observed to directly go to water sources for meeting their drinking needs; therefore, water in food is not crucial for them. Individuals of the species *G. beringei* are also known to drink from streams to meet their water requirements (Rothman et al., 2008). Therefore, our results suggest that Tibetan macaques may be inclined to choose foods with low fiber and tannin content and water may not be a limiting factor in their food choices.

Surprisingly, our study found that protein was excluded from all highly supported models and there was no difference between food and non‐food. This does not support our predictions (iii). This is different from other studies, for example, in which *L. leucopus* (Dröscher et al., 2016) and *M. assamensis* (Li et al., 2020) opted for high‐protein foods. Studies have shown that once protein concentrations exceed the requirements, food choices may be based on other components or criteria (Ganzhorn et al., 2017). In the present study, Tibetan macaques were found to have a fruit‐dominated diet (52.5%). In different seasons, they showed significant dependence on some key species. The main food varieties are rich and varied, including bamboo shoots (protein percentages: 36.50%) of *Phyllostachys edulis*, fruits (protein percentages: 0.52%) and leaves (protein percentages: 0.42%) of *Eurya japonica*, fruits (protein percentages: 0.70%) of *Stauntonia brachyanthera*, and fruits (protein percentages: 3.90%) of *Castanopsis eyrei*. Proteins are more restricted in protein‐deficient environments. In other words, Tibetan macaques can easily get the protein they need from a variety of foods, without having to seek out high‐protein foods.

Fat, sugar, and starch are important sources of energy (National, 2003). *G. gorilla gorilla*, *P. troglodytes*, White‐bellied spider monkeys (*Ateles belzebuth*), and *N. nasutus*, all vary in size but enjoy a diet high in sugar, especially ripe fruit (Ma et al., 2017; Remis, 2002; Stevenson & Link, 2010), while *A. pigra* (Righini et al., 2015) opts for high‐fat foods. What was different in our study was that there were no significant differences in fat, total sugar, and starch content between Tibetan macaques' staple foods and non‐food. Although total sugar and starch were included in the highly supported models (ΔAICc ≤ 2; Table 6), their model‐averaged 95% confidence interval of the regression coefficient (*β*) contained zero. This is not consistent with our prediction (iv). Species richness and resource availability are high in the low‐altitude habitats of Tibetan macaques (Li et al., 2021). Abundant food resources in low‐altitude habitats give Tibetan macaques great freedom in food choice. They can get enough food to satisfy their body's needs with minimal expenditure instead of seeking out foods high in fat and sugar. As a result, they can meet their needs while avoiding excessive intake of nutrients such as tannins and fiber that are bad for digestion and absorption. The low‐fiber and low‐tannin foraging strategy may be an adaptation for Tibetan macaques to the abundance of resources in low‐altitude habitats.

In summary, our study found that the foraging behavior of Tibetan macaques is highly flexible and adaptable. They change their foraging strategies in response to environmental changes both spatially and temporally. Tibetan macaques living at low altitudes prefer low‐fiber and low‐tannin foods. This diverse dietary preference adapts to higher species richness in low‐altitude habitats and may provide the energy necessary for Tibetan macaques to maintain their large body size. Our findings confirmed the effect of nutrients on food choice in Tibetan macaques. We also demonstrated the complexity of primate foraging behavior in terms of nutrition, food availability, and geographical distribution. The diet of primates should be defined comprehensively rather than simply as folivore or frugivore. There were some limitations in this study. Due to the lack of individual identification of wild Tibetan macaques, the instantaneous scanning sampling method adopted in this study could not quantify the nutritional intake of macaques and the foraging data of macaques at high altitudes in the same region were lacking. Although we uncovered some of the nutritional foraging strategies of macaques, quantitative analysis is the next step. Our next goal is to analyze nutrient intake based on individual identification and collect high‐altitude foraging data. As the nutrient balance hypothesis suggests, animals balance their nutrition by mixing different foods with different nutrients, rather than maximizing the availability of one nutrient (Raubenheimer & Simpson, 2004). Due to their large body size, Tibetan macaques have great nutritional and energy requirements. They may prefer a balanced intake of nutrients rather than relying on a single nutrient. Therefore, nutritional balance strategy will also be one of our future research directions. We will further quantitatively analyze the nutritional strategies of Tibetan macaques to fully reveal the adaptive strategies of their foraging behavior.

## AUTHOR CONTRIBUTIONS

**Bowen Li:** Data curation (lead); formal analysis (equal); investigation (lead); methodology (equal); visualization (equal); writing – original draft (lead); writing – review and editing (lead). **Wen‐Bo Li:** Investigation (supporting); writing – review and editing (supporting). **Chao Liu:** Investigation (supporting); writing – review and editing (supporting). **Peipei Yang:** Investigation (supporting); writing – review and editing (supporting). **Jin Hua Li:** Conceptualization (lead); funding acquisition (lead); resources (lead); supervision (equal); visualization (equal); writing – review and editing (equal).

## CONFLICT OF INTEREST

None declared.

## Data Availability

The dataset generated and analyzed during the current study is available in the open figshare repository https://doi.org/10.6084/m9.figshare.19248042.
